# Cold Baths in Typhoid Fever

**Published:** 1890-02

**Authors:** 


COLD BATHS IN TYPHOID FEVER.—It has been shown by the statistics of Josias, Jehel-Renay and Richard, that by giving the patients a bath every three hours, when their temperature exceeded 102 degrees, that the mortality was only 4:61 per cent: whereas in the ordinary treatment it was 15 per cent:

